# Longitudinal Associations between Perceived Quality of Living Spaces and Health-Related Quality of Life among Homeless and Vulnerably Housed Individuals Living in Three Canadian Cities

**DOI:** 10.3390/ijerph16234808

**Published:** 2019-11-29

**Authors:** Carly Magee, Monica Norena, Anita M. Hubley, Anita Palepu, Stephen W. Hwang, Rosane Nisenbaum, Mohammad Ehsanul Karim, Anne Gadermann

**Affiliations:** 1 School of Population and Public Health, University of British Columbia, Vancouver, BC V6T 1Z3, Canada; ehsan.karim@ubc.ca (M.E.K.); anne.gadermann@ubc.ca (A.G.); 2 Human Early Learning Partnership, University of British Columbia, Vancouver, BC V6T 1Z3, Canada; 3 Centre for Health Evaluation and Outcome Sciences, St. Paul’s Hospital, Vancouver, BC V6T 1Z3, Canada; mnorena@hivnet.ubc.ca (M.N.); apalepu@hivnet.ubc.ca (A.P.); 4 Department of Educational and Counselling Psychology, and Special Education, University of British Columbia, Vancouver, BC V6T 1Z4, Canada; anita.hubley@ubc.ca; 5 Division of General Internal Medicine, Department of Medicine, University of British Columbia, Vancouver, BC V6T 1Z3, Canada; 6 MAP Centre for Urban Health Solutions, Li Ka Shing Knowledge Institute, St. Michael’s Hospital, University of Toronto, Toronto, ON M5B 1A6, Canada; stephen.hwang@unityhealth.to (S.W.H.); rosane.nisenbaum@unityhealth.to (R.N.); 7 Applied Health Research Centre, Li Ka Shing Knowledge Institute, St Michael’s Hospital, Toronto, ON M5B 1A6, Canada; 8 Dalla Lana School of Public Health, University of Toronto, Toronto, ON M5T 3M7, Canada

**Keywords:** homeless persons, vulnerably housed, housing, health, health-related quality of life

## Abstract

The objective of this study was to examine longitudinal associations between perceived quality of living spaces and mental and physical health-related quality of life (HRQoL) among homeless and vulnerably housed individuals living in three Canadian cities. The Health and Housing in Transition (HHiT) study was a prospective cohort study conducted between 2009 and 2013 of N = 1190 individuals who were homeless and vulnerably housed at baseline. Perceived quality of living spaces (based on rated comfort, safety, spaciousness, privacy, friendliness and overall quality) and both mental and physical HRQoL were assessed at baseline and at four annual follow up points. Generalized estimating equation (GEE) analyses were used to examine associations between perceived quality of living spaces and both mental and physical HRQoL over the four-year study period, controlling for time-varying housing status, health and socio-demographic variables. The results showed that higher perceived quality of living spaces was positively associated with mental (*b* = 0.42; 95% CI 0.38—0.47) and physical (*b* = 0.11; 95% CI 0.07—0.15) HRQoL over the four-year study period. Findings indicate that policies aimed at increasing HRQoL in this population should prioritize improving their experienced quality of living spaces.

## 1. Introduction

Homelessness is a major public health concern in Canada, with at least 235,000 individuals estimated to experience homelessness annually [1]. Moreover, an even larger number of individuals are vulnerably housed, living in precarious or sub-standard housing [2,3]. In Canadian cities, homeless and vulnerably housed individuals are concentrated in low-income, urban areas, where the majority of shelters, marginal housing, and associated health and social services are located [4,5]. Research with homeless and vulnerably housed individuals living in these areas has found that experiences of being either homeless or housed tend to be transitory, rather than stable life contexts [6]. Moreover, prior work has found that distinctions between being homeless as opposed to housed are less salient among populations accessing low-quality, socially marginal housing [7]. Research has found that both homeless individuals and vulnerably housed individuals living in marginal housing (e.g., single room occupancy hotels and rooming houses) experience worse physical and mental health and increased mortality rates [4,8,9,10]. For example, Hwang et al. [9] found that life expectancy was reduced by 13 and eight years for men and women living in shelters, 11 and nine years for men and women living in rooming houses, and eight and five years for men and women living in single room occupancy hotels, respectively, compared to the general population. 

An important component of health among homeless and vulnerably housed individuals is health-related quality of life (HRQoL). HRQoL measures individuals’ perception of how they are impacted by their mental and physical health [11]. This reflects the World Health Organization’s definition of health as “a state of complete physical, mental and social well-being and not merely the absence of disease or infirmity” [12]. In addition, studies have shown that HRQoL is predictive of morbidity and mortality [13,14]. Similar to other measures of health, both mental and physical HRQoL have been shown to be reduced among homeless and vulnerably housed individuals, compared to the general population [4,15]. In addition, the baseline results from the health and housing in transition (HHIT) cohort study in three Canadian cities found that homeless and vulnerably housed individuals did not differ greatly on both mental and physical HRQoL [4]. 

Given the negative health consequences associated with being both homeless and vulnerably housed, and the importance of HRQoL, it is critical to understand the predictors of both mental and physical HRQoL in this population. Prior research has identified demographic, health, and social support variables that are predictive of either or both physical and mental HRQoL among homeless and vulnerably housed individuals [16,17,18]. For instance, Cherner et al. [16] found that among homeless and vulnerably housed adults in Vancouver, Toronto and Ottawa, having fewer chronic health conditions and greater social support was associated with higher mental HRQoL among both men and women. In the current study, we investigated the extent to which homeless and vulnerably housed individuals’ perceptions of the quality of their living spaces is associated with their physical and mental HRQoL.

The quality of living spaces may impact HRQoL through a number of pathways. Lower quality physical conditions of living spaces increase the likelihood of exposure to environmental hazards (e.g., poor ventilation, mold, and low heat) that are associated with ill health [7,19], which in turn is associated with lower HRQoL [13]. In addition, the social climate of a living space may positively benefit HRQoL through facilitating the development of supportive relationships and connections to health and social services [7,19]. Furthermore, individuals’ experience of their living spaces as having positive qualities (e.g., privacy, safety, and friendliness) may benefit HRQoL by contributing to psychological well-being and reducing stress [7,19].

Past research has demonstrated that the physical conditions of housing are associated with health status and HRQoL [3,19,20,21,22,23]. For instance, Hwang et al. [22] had observers rate the physical conditions of rooming houses (e.g., building structure, noise levels) and found that individuals living in the lowest quality rooming houses reported the lowest levels of both mental and physical HRQoL (measured with the short form health survey; SF-36). In addition, a select number of studies have examined associations between perceived social and physical qualities of living spaces and HRQoL or general quality of life [24,25,26]. For example, Rourke et al. [24] found that satisfaction with both material (e.g., light, heating and air quality of dwelling) and meaning (e.g., feelings of identity, control and belonging associated with dwelling) dimensions of housing were associated with both mental and physical HRQoL among 519 individuals living with HIV (human immunodeficiency virus) in Ontario. It is important to examine individuals’ perception of the quality of their living spaces, as this reflects their psychological experience of these spaces.

Much of the existing research examining associations between the perceived quality of living spaces and HRQoL has relied on cross-sectional designs or longitudinal designs with follow-up periods of one year or less [24,26]. In addition, the majority of research examining associations between the quality of living spaces and HRQoL has focused on housed individuals. However, there is substantial variation in the types of spaces that homeless individuals occupy (e.g., sheltered spaces, outdoor spaces, and couch surfing) and this variation may have important consequences for HRQoL [27]. For instance, one study [28] conducted with a sample of 209 caregivers living in homeless shelters in New York City found that negative perceptions of the social environment of shelters were associated with worse mental health. Very little research has examined the perceived quality of living spaces and HRQoL among homeless individuals.

In sum, it is important to examine predictors of HRQoL in homeless and vulnerably housed individuals as this is a key component of health [11,12,13,14] that has been shown to be reduced in this population [4,15]. Moreover, it is vital to examine individuals’ perception of the social and physical qualities of their living spaces as this reflects their personal experience of these spaces, which has the potential to uniquely affect HRQoL [7,19]. While past research has demonstrated that housing quality is associated with health [19,20,21,22,23], and that perceived housing quality is associated HRQoL in specific populations [24], few studies have examined associations between perceived quality of living spaces and both mental and physical HRQoL in diverse samples of homeless and vulnerably housed individuals. To address these gaps, we examined the association between perceived quality of living spaces (based on rated comfort, safety, spaciousness, privacy, friendliness and overall quality) and both mental and physical HRQoL among individuals in Vancouver, Toronto and Ottawa who were either homeless or vulnerably housed at baseline. Moreover, we examined these associations between perceived quality of living spaces and both mental and physical HRQoL over a four-year follow-up period and controlled for time-varying housing status and several time-varying and fixed clinical and demographic variables. Given past research demonstrating connections between perceived housing quality and HRQoL among individuals with HIV [24] and between perceived homeless shelter social environment and mental health [28], we expected that perceived quality of living spaces would be positively associated with both mental and physical HRQoL among individuals who were homeless and vulnerably housed at baseline in our study.

## 2. Materials and Methods

### 2.1. Sample and Procedure

The Health and Housing in Transition (HHiT) study was a prospective cohort study of homeless and vulnerably housed individuals from 2009 to 2013. The objective of the HHIT study was to examine health and housing status over time among homeless and vulnerably housed individuals. Participants were single adults aged 18 and over living in one of three Canadian cities (Vancouver, Ottawa and Toronto). At baseline, the sample was comprised of 595 homeless and 595 vulnerably housed individuals, with equal numbers of homeless and vulnerably housed individuals from each city. Individuals were considered homeless if they were living in a shelter, public space, vehicle, abandoned building, or in someone else’s home in the past week. Individuals were considered to be vulnerably housed if they reported living in their own room or unit but had either moved at least two times or had been homeless in the previous year. 

Participants were recruited for this study between January and December 2009. Homeless participants were recruited at shelters and meal programs, whereas vulnerably housed individuals were recruited at rooming houses, single room occupancy hotels, and meal programs. Data were collected using in-person, structured interviews at five time points: baseline and at 1, 2, 3, and 4 years follow up. Interviews were conducted by trained research personnel and typically lasted 60–90 min. Follow-up rates over the four-year period ranged from 79.2% to 83.7%. Losses to follow-up were due to participant deaths, refusals or withdrawals, and inability to contact or locate participants. All participants provided informed consent and were compensated $20 CAD at the time of each interview. This research was conducted in accordance with the Declaration of Helsinki and approved by the Research Ethics Boards at the University of British Columbia (project identification code = H08-00585; date of approval = 16 May 2008), the University of Ottawa (project identification code = 04-08-09; date of approval = 10 July 2008), and St. Michael’s Hospital in Toronto (project identification code = REB08-014c; date of approval = 24 March 2008). A detailed description of the HHiT study design and recruitment methodology can be found in Hwang et al. [4].

### 2.2. Measures

Health-related quality of life (HRQoL), perceived quality of living spaces, housing status, problematic substance use, and time were measured at all five time points. Demographics, chronic health conditions and mental health conditions were measured at baseline only.

#### 2.2.1. Outcome Variables

Health-related quality of life (HRQoL). HRQoL was measured with the Short Form Health Survey Version 1 (SF-12) at all five time points. The SF-12 [29] is a shortened version of the SF-36 [30] designed to reproduce the Physical Component Summary (PCS) and a Mental Component Summary (MCS) scores. The standardized summary scores, based on United States population norms, have a mean of 50 and SD of 10. Higher scores reflect higher HRQoL. Previous research has provided evidence for the construct validity of the SF-12 in diverse samples, including individuals with severe mental illness and individuals who are homeless [31,32]

#### 2.2.2. Primary Explanatory Variable

Perceived Quality of Living Spaces. Perceived quality of living spaces was assessed at all five time points with Toro’s Housing Quality Instrument [33]. This scale is comprised of 6 items that ask participants to rate the place where they currently live in terms of comfort, safety, spaciousness, privacy, friendliness and overall quality on a scale from 1 (“very bad”) to 7 (“very good”). Ratings on each item were summed, and total scores ranged from 6–42, with higher scores indicating the better perceived quality of living space. This measure was designed in such a way that participants could respond irrespective of their housing status and has been shown to have good test-retest reliability [34]. 

#### 2.2.3. Covariates 

Housing Status. Housing status was assessed at all five time points. Individuals were categorized as being homeless or housed (coded as homeless = 1 and housed = 0) using the residential timeline follow-back inventory [35]. Individuals were considered housed if they were living in their own apartment or house, living with friends or family and paying rent, living in a rooming house, nursing home, or in supportive housing. They were considered to be homeless if they were living in a homeless shelter, living on the streets, campground, motel or hotel. Time spent in institutions (e.g., prison, substance abuse treatment facility) was categorized as either homeless or housed using the methodology described elsewhere https://tspace.library.utoronto.ca/handle/1807/69938. At baseline, vulnerably housed and homeless individuals met criteria for being housed and homeless, respectively. 

Problematic substance use. Problematic substance use in the past 12 months was assessed at all five time points and was coded as yes = 1 and no = 0. The 10 item Alcohol Use Disorders Identification Test (AUDIT) was used to screen for alcohol use disorder, with a score of ≥20 indicating problematic alcohol use [36]. An example item for the AUDIT is: “During the past 12 months, how many drinks containing alcohol did you have on a typical day?” (Response categories: 1–2; 3–4; 5–6; 7–9; 10 or more). The 10 item Drug Abuse Screening test (DAST-10) was used to screen for problematic drug use, with a score of ≥6 indicating problematic drug use [37]. An example item for the DAST-10 is: “in the past 12 months were you always able to stop drugs when you wanted to? (coded as yes = 1 and no = 0). Thus, problematic substance use was defined as having a DAST-10 score of ≥6 and/or an AUDIT score of ≥20 in the past 12 months [38]. Prior research has demonstrated the validity and reliability of the AUDIT and DAST-10 for measuring problematic substance use in vulnerable populations [39,40]. 

Time. Time was a continuous variable indicating the year of data collection and ranged from 1 to 5, representing baseline and the four annual follow up time points. 

Demographics. Demographics were recorded at baseline only. Participants self-reported their age, gender (male, female or transgender), ethnicity (White, Black/African Canadian, Indigenous, and other) and city of residence (Toronto, Ottawa, Vancouver). Information about the specific gender identity of transgender participants (i.e., male, female, non-binary, and other) was not collected. We acknowledge that classifying all transgender individuals as the same is an over-simplification of the gender identities of these individuals.

Chronic health conditions. Chronic health conditions were recorded at baseline only. Participants self-reported on chronic health conditions (lasting at least six months and diagnosed by a health care professional) using items from the Canadian Community Health Survey [41]. Specifically, participants were presented with a list of 32 chronic health conditions (e.g., cardiovascular, respiratory, gastrointestinal, musculoskeletal, neurological, sensory, and emotional disorders) and asked whether they had been diagnosed with any of these conditions by a medical professional and if the condition had lasted at least six months. The presence of three or more chronic health conditions was coded as yes = 1 and no = 0. The presence of three or more chronic health conditions has been used in prior research on homeless and vulnerably housed populations to identify individuals with a heavier burden of chronic health problems [4]. 

Mental Health Condition. Diagnosis with mental health conditions was recorded at baseline only. Participants were asked whether they had ever been diagnosed with a mental health problem; this was coded as yes = 1 and no = 0.

### 2.3. Analyses

Descriptive statistics (means and standard deviations for continuous variables and frequencies and percentages for categorical variables) were calculated for all study variables measured at baseline. In addition, mean perceived quality of living spaces, and the respective standard deviations, were calculated separately for the baseline sample of homeless and vulnerably housed individuals. Moreover, a *t*-test was conducted to examine whether any observed difference in mean perceived quality of living spaces between homeless and vulnerably housed individuals at baseline was statistically significant. Next, generalized estimating equation (GEE) analyses were used to estimate population average associations between predictor variables (perceived quality of living spaces and covariates) and outcome variables (physical and mental HRQoL) over the four-year study period. GEE modelling is a method used to estimate population average associations between variables that may vary over time, while accounting for the fact that observations are nested within individuals. Dependencies in the data due to repeated measures on individuals are accounted for by using an exchangeable correlation structure to obtain estimates of standard errors [42]. 

Age, gender, city of residence, having three or more chronic health conditions, and ever having been diagnosed with a mental health condition were entered into the GEE models as fixed variables. By contrast, perceived quality of living spaces, problematic substance use, housing status (i.e., being homeless versus housed at each time point), and time were entered as time-varying variables. These variables were measured at all five time points and were allowed to vary over time in GEE models. Unadjusted and adjusted population average associations between predictors and outcomes were estimated using bivariable and multivariable GEE models, respectively. In adjusted models, the interaction between perceived quality of living spaces and housing status in predicting both mental and physical HRQoL was tested. That is, we examined whether the association between perceived quality of living spaces and both mental and physical HRQoL differed depending on whether individuals were homeless or housed at each time point. Non-significant interaction terms were dropped from the final models. We reported model regression coefficients with 95% confidence intervals and considered *p*-values of less than 0.05 statistically significant. For multivariate models 7% of observations were excluded due to missing data on one or more study variables. All analyses were performed in SAS (9.2, http://www.sas.com/).

## 3. Results

### 3.1. Description of Sample

Baseline descriptive statistics for all study variables are reported in Table 1. Half of the study sample was homeless (as opposed to housed) at baseline. Approximately two-thirds of the sample were male, one-third female, and less than 2% identified as transgender. Participants were on average 42.2 years old, 62% White, 9.2% Black/African Canadian, 17.6% Indigenous, and 10.7% were of another ethnicity. Approximately one-third of the sample resided in Ottawa, Vancouver and Toronto (33.3%, 33.2, 33.5%, respectively). Mean perceived quality of living spaces was 27 (SD = 8.2) overall, and 26.7 (SD = 8.0) for homeless individuals and 27.4 (SD = 8.3) for housed individuals at baseline. A *t*-test revealed that mean perceived quality of living spaces was not significantly different between homeless and housed individuals (*t* = 1.43 (*df* = 589), *p* = 0.37).

### 3.2. GEE Model Results

Table 2 shows the unadjusted and adjusted regression coefficients estimated from the bivariable and multivariable GEE analyses. The results showed that in the unadjusted model, perceived quality of living spaces was positively associated with both mental HRQoL (b = 0.47; 95% CI: 0.43, 0.51) and physical HRQoL (b = 0.10; 95% CI: 0.06, 0.14). In the adjusted model, perceived quality of living spaces remained significantly and positively associated with mental HRQoL (b = 0.42; 95% CI: 0.38, 0.47) and physical HRQoL (b = 0.11; 95% CI: 0.07, 0.15), while controlling for housing status at each interview point (being in a state of homeless versus housed), age, gender, ethnicity, city of residence, whether individuals had ever been diagnosed with a mental health condition, whether individuals had three or more chronic health conditions, whether individuals reported problematic substance use in the past 12 months, and time. Interaction terms between perceived quality of living spaces and housing status (homeless versus housed) were not significant in predicting mental (*p* = 0.58) or physical (*p* = 0.07) HRQoL. As such, these terms were not retained in the final models.

When examining the associations between the covariates and mental HRQoL in the unadjusted (bivariable) model, being younger, being female (compared to being male), ever being diagnosed with a mental health condition, having three or more chronic health conditions, and reporting problematic substance use in the past 12 months was associated with lower mental HRQoL. In the adjusted (multivariable) model, similar results were found except that being in a homeless state (compared to being in a housed state) rather than being younger was associated with lower mental HRQoL. There were no statistically significant differences in mental HRQoL by ethnicity, city of residence, or for transgender individuals in unadjusted and adjusted models.

When examining the associations between the covariates and physical HRQoL in the unadjusted (bivariable) model, being older, being female (compared to being male), ever being diagnosed with a mental health condition and having three or more chronic health conditions, was associated with lower physical HRQoL. In addition, reporting Black/African Canadian ethnicity (compared to reporting White ethnicity) was associated with higher physical HRQoL. In the adjusted (multivariable) model, similar results were found except having Black/African Canadian ethnicity was no longer significantly associated with physical HRQoL and reporting problematic substance use in the past 12 months was significantly associated with lower physical HRQoL. There were no statistically significant differences in physical HRQoL by being in a homeless state (compared to being in a housed state), ethnicity, city of residence, or for transgender individuals (compared to males) in unadjusted and adjusted models. Time was positively associated with mental HRQoL and negatively associated with physical HRQoL in both unadjusted and adjusted models, such that individuals’ mental HRQoL tended to improve, and their physical HRQoL tended to worsen, over time.

## 4. Discussion

Understanding the predictors of HRQoL among homeless and vulnerably housed individuals is important, as HRQoL is a key component of health predictive of morbidity and mortality [11,12,13,14] and because there are over 200,000 homeless and over 600,000 vulnerably housed individuals living in Canada [1,2]. In the current study, our key finding was that, over time, both higher mental and physical HRQoL were associated with more positive perceptions of one’s living spaces, as reported by a sample of individuals who were homeless and vulnerably housed at baseline. Notably, these associations were observed over a four-year follow-up period and while controlling for covariates such as time-varying housing status (homeless or housed at each time point) as well as several fixed and time-varying clinical health and socio-demographic variables associated with HRQoL. Moreover, we found that the perceived quality of living spaces did not differ between homeless and vulnerably housed individuals at baseline, and that the longitudinal association between perceived quality of living spaces and HRQoL was not moderated by time-varying housing status (homeless versus housed at each time point).

Findings from this study align with previous research demonstrating a connection between housing quality and health [19,20,21,22,23], perceived housing quality and general HRQoL among specific populations [24], and perceived social environment of a homeless shelter and mental health [28]. In addition, the current study extends prior work by demonstrating that perceived quality of living spaces is positively associated with both mental and physical HRQoL in a diverse sample of homeless and vulnerably housed individuals in three cities in Canada. Moreover, by examining associations over a four-year follow up period, this study provided more reliable estimates of population average associations between perceived quality of living spaces and both mental and physical HRQoL, compared to previous studies that were either cross-sectional or based on shorter and fewer follow up periods.

The findings from this study that individuals who were homeless and vulnerably housed at baseline did not differ in their report of the quality of their living spaces, and that the longitudinal associations between perceived quality of living spaces and physical and mental HRQoL did not differ by time-varying housing status, aligns with prior research findings that distinctions between being housed and homeless are less salient among homeless individuals and vulnerably housed individuals living in highly marginalized, low-quality housing [4,7]. Particularly in this population, housing status does not fully capture individuals experience of their living spaces. Perceived social and physical qualities of living spaces vary for both homeless and housed individuals in these socially marginal areas, and someone who is homeless may have more positive perceptions of their living spaces compared to someone who is housed. For instance, a homeless person living in a tent city may feel safer and more socially connected compared to someone living in a single room occupancy hotel or rooming house [7,43].

These findings indicate that housing policy should prioritize access to high-quality housing that takes into consideration individuals’ subjective experience of their living spaces, in addition to their health care needs and the physical conditions of their living spaces. A focus on housing individuals without thoughtful consideration of quality and subjective experience, such as forcibly removing people from tents on city property and offering limited spaces in single room occupancy hotels and shelters [44], is likely to miss opportunities to improve HRQoL. Moreover, findings from this study indicate that improving the experienced social and physical quality of shelters may be associated with improved HRQoL among currently homeless individuals.

This study has a number of limitations. First, the use of observational data restricts our ability to determine causality. While the higher perceived quality of living spaces may improve HRQoL, it may also be the case that higher HRQoL leads to more favorable perceptions of the quality of living spaces, or that more optimistic individuals perceive both higher quality living spaces and higher HRQoL. Future research may better assess the causality of these associations by experimentally improving living conditions and measuring the effects on the perceived quality of living spaces and HRQoL and controlling for personal characteristics such as optimism. Second, this study did not include objective measures of the physical conditions of living spaces. As such, it was not possible to tease apart the individual effects of the subjective experience of quality and the objective physical conditions of living spaces. Future research should address this by obtaining both objective and subjective measures of the quality of living spaces and specific physical characteristics of living spaces. This would also allow for an examination of the ways in which specific physical characteristics of living spaces may be associated with an improved subjective experience of quality. Finally, because this research focused on vulnerably housed individuals living in specific forms of marginal housing (i.e., single room occupancy hotels and rooming houses) and homeless individuals who accessed shelters and meal programs, the results may not generalize to other homeless and vulnerably housed populations who live in different forms of housing or do not access the service sites sampled.

## 5. Conclusions

We found that among individuals who were homeless and vulnerably housed at baseline, higher perceived quality of living spaces was associated with both greater mental and physical HRQoL over a four-year study period, even after controlling for time-varying housing status and several clinical health and socio-demographic variables associated with HRQoL. Findings from this study indicate that policy aimed at increasing HRQoL among homeless and vulnerably housed individuals should prioritize improving their experienced quality of living spaces.

## Figures and Tables

**Table 1:** Descriptive Statistics of Study Variables at Baseline (n = 1190).

Perceived Quality of Living Spaces, mean (SD)	27 (8.2)
Homeless, n (%)	599 (50.4)
Age, mean (SD)	42.2 (10.6)
Gender, n (%)	
Female	385 (32.3)
Male	788 (66.2)
Transgender	17 (1.4)
Ethnicity, n (%)	
White	723 (62.6)
Black/African Canadian	106 (9.2)
Indigenous	203 (17.6)
Other	123 (10.7)
City, n (%)	
Toronto	399 (33.5)
Ottawa	396 (33.3)
Vancouver	395 (33.2)
Ever diagnosed MH problem, n (%)	605 (52)
Three or more chronic health conditions, n (%)	593 (50)
Problematic Substance use, n (%)	444 (37)
SF-12 physical component score, mean (SD)	44.52 (11.28)
SF-12 mental component score, mean (SD)	39.11 (13.03)

MH: mental health.

**Table 2:** Unadjusted and Adjusted Associations between Perceived Quality of Living Spaces and Both Mental and Physical HRQoL.

	Mental Component Summary (MCS)-12		Physical Component Summary (PCS)-12	
	Unadjusted	Adjusted	Unadjusted	Adjusted
Perceived Quality of Living Spaces *	0.47 (0.43, 0.51)	0.42 (0.38, 0.47)	0.10 (0.06, 0.14)	0.11 (0.07, 0.15)
Homeless *	−0.85 (−1.95, 0.26)	−1.46 (−2.27, −0.66)	0.77 (−0.29, 1.84)	0.69 (0.00, 1.38)
Age (per decade)	0.63 (0.09, 1.17)	0.10 (−0.38, 0.59)	−2.38 (−2.87, −1.89)	−1.93 (−2.39, −1.47)
Gender				
Male	Reference	Reference	Reference	Reference
Female	−2.53 (−3.7, −1.36)	−2.06 (−3.13, −0.99)	−3.02 (−4.16, −1.88)	−1.78 (−2.81, −0.74)
Transgender	−1.70 (−5.96, 2.57)	0.52 (−2.84, 3.88)	−0.27 (−4.77, 4.23)	−0.8 (−5.4, 3.8)
Ethnicity				
White	Reference	Reference	Reference	Reference
Black/African Canadian	1.92 (−0.02, 3.86)	1.14 (−0.79, 3.08)	2.64 (0.78, 4.5)	−0.01 (−1.67, 1.64)
Indigenous	−0.07 (−1.53, 1.39)	0.71 (−0.56, 1.97)	−1.31 (−2.7, 0.09)	0.26 (−1.0, 1.52)
Other	−0.32 (−2.19, 1.55)	−0.16 (−1.79, 1.47)	0.38 (−1.38, 2.14)	−0.93 (−2.38, 0.53)
City				
Toronto	Reference	Reference	Reference	Reference
Ottawa	−0.66 (−2.02, 0.69)	0.96 (−0.25, 2.17)	−0.49 (−1.81, 0.84)	0.31 (−0.89, 1.50)
Vancouver	−0.46 (−1.77, 0.85)	0.84 (−0.34, 2.03)	−1.22 (−2.47, 0.02)	0.03 (−1.10, 1.17)
Ever diagnosed MH condition	−5.44 (−6.51, −4.38)	−4.47 (−5.48, −3.45)	−2.94 (−4.00, −1.88)	−1.51 (−2.47, −0.56)
Three or more chronic health conditions	−2.97 (−4.06, −1.87)	−1.61 (−2.64, −0.57)	−9.16 (−10.09, −8.23)	−7.65 (−8.67, −6.63)
Problematic substance use *	−4.76 (−5.84, −3.67)	−4.41 (−5.18, −3.64)	−0.06 (−1.15, 1.02)	−0.82 (−1.5, −0.13)
Time	1.04 (0.82, 1.25)	0.44 (0.23, 0.66)	−0.26 (−0.44, −0.09)	−0.27 (−0.45, −0.09)

Note. * indicates time varying. Bolded numbers indicate that the 95% confidence interval does not cross 0. Ever diagnosed mental health condition (yes = 1; no = 0). Three or more chronic health conditions (yes = 1; no = 0). Problematic substance use (yes = 1; no = 0). Homeless (yes = 1; no = 0).
